# Pro-poor policies and improvements in maternal health outcomes in India

**DOI:** 10.1186/s12884-021-03839-w

**Published:** 2021-05-19

**Authors:** M. Bhatia, L. K. Dwivedi, K. Banerjee, A. Bansal, M. Ranjan, P. Dixit

**Affiliations:** 1 Department of Health Policy, London School of Economics and Political Science, Houghton Street, London, WC2A 2AE UK; 2 International Institute for Population Sciences, Mumbai, India; 3 Department of Statistics, Mizoram University, Pachhunga University College Campus, Aizawl, Mizoram India; 4 School of Health Systems Studies, Tata Institute of Social Sciences, Mumbai, India

**Keywords:** Maternal health, Maternal mortality, Maternal health outcomes, Financial incentives, Conditional cash transfers, Demand side financing, Developing countries, India

## Abstract

**Background:**

Since 2005, India has experienced an impressive 77% reduction in maternal mortality compared to the global average of 43%. What explains this impressive performance in terms of reduction in maternal mortality and improvement in maternal health outcomes? This paper evaluates the effect of household wealth status on maternal mortality in India, and also separates out the performance of the Empowered Action Group (EAG) states and the Southern states of India. The results are discussed in the light of various pro-poor programmes and policies designed to reduce maternal mortality and the existing supply side gaps in the healthcare system of India. Using multiple sources of data, this study aims to understand the trends in maternal mortality (1997–2017) between EAG and non EAG states in India and explore various household, economic and policy factors that may explain reduction in maternal mortality and improvement in maternal health outcomes in India.

**Methods:**

This study triangulates data from different rounds of Sample Registration Systems to assess the trend in maternal mortality in India. It further analysed the National Family Health Surveys (NFHS). NFHS-4, 2015–16 has gathered information on maternal mortality and pregnancy-related deaths from 601,509 households. Using logistic regression, we estimate the association of various socio-economic variables on maternal deaths in the various states of India.

**Results:**

On an average, wealth status of the households did not have a statistically significant association with maternal mortality in India. However, our disaggregate analysis reveals, the gains in terms of maternal mortality have been unevenly distributed. Although the rich-poor gap in maternal mortality has reduced in EAG states such as Bihar, Odisha, Assam, Rajasthan, the maternal mortality has remained above the national average for many of these states. The EAG states also experience supply side shortfalls in terms of availability of PHC and PHC doctors; and availability of specialist doctors.

**Conclusions:**

The novel contribution of the present paper is that the association of household wealth status and place of residence with maternal mortality is statistically not significant implying financial barriers to access maternal health services have been minimised. This result, and India’s impressive performance with respect to maternal health outcomes, can be attributed to the various pro-poor policies and cash incentive schemes successfully launched in recent years. Community-level involvement with pivotal role played by community health workers has been one of the major reasons for the success of many ongoing policies. Policy makers need to prioritise the underperforming states and socio-economic groups within the states by addressing both demand-side and supply-side measures simultaneously mediated by contextual factors.

**Supplementary Information:**

The online version contains supplementary material available at 10.1186/s12884-021-03839-w.

## Background

It is estimated that nearly 303,000 women die per year from maternal causes with almost all of these deaths occurring in low-resource settings [1, 2]. Given that most of these deaths can be easily prevented or treated with cheap and effective interventions, such high maternal mortality and morbidity is unacceptable even in resource constrained settings. It is therefore not surprising that reducing maternal mortality and morbidity has been an important concern for both national governments and international organizations. The risk of a woman dying as a result of pregnancy or childbirth during her lifetime is about one in six in the poorest parts of the world compared with about one in 30,000 in Northern Europe [3]. Such inequalities poses a huge challenge to meeting the post Millennium Development Goals (MDGs) in the era of Sustainable Development Goals (SDGs).

India is the world’s largest democratic nation, with 16% of the global population. Unfortunately, India has the highest number of maternal deaths in the world, contributing a total of 45,000 maternal deaths in 2015 [4]. It is one of the six countries that contributes to 50% of the world’s maternal mortality [4]. Healthcare in India is the responsibility of individual states, which vary in terms of their level of socio-economic development, size of population, experience of epidemiological transition, and health system capacities, factors which influence the health status experienced by the population of the states. On one hand, states such as Kerala experience relatively low levels of maternal mortality which are comparable with developed countries, whereas a few of the states belonging to Empowered Action Group (EAG) (a group of socio-economically backward states), such as Madhya Pradesh and Uttar Pradesh suffer high maternal mortality comparable with some of poorest countries of the world [5]. Reduction in maternal mortality in India has been a very slow process but much more rapid decline has been observed in recent years.

A number of countries have adopted various strategies that have contributed to the decline in maternal mortality over the years, from a single intervention to a complex set of public health approaches like safe motherhood strategies promoted by WHO, and UNICEF [6]. These include strategies like ANC, delivery by trained personnel, promotion of institutional delivery, and access to emergency obstetric services by strengthening health systems, and addressing broader social determinants of maternal health. In addition, many countries are experimenting with demand side financing initiatives including the role of conditional cash transfers to reduce financial barriers in accessing maternal health services (8–16).

India’s achievements in reduction of maternal mortality in recent years can be considered a success story as maternal mortality has declined over the years from 556 per 1000 live births in 1990 to 174 in 2015 at a rate of 15.8% annually [7]. Since 2005, India has experienced an impressive 77% reduction in maternal mortality compared to the global average of 43% [8]. In fact, this achievement has not gone unnoticed and India has been commended for this remarkable feat of drastically reducing maternal mortality by the WHO [8]. What explains this impressive performance in terms of reduction in maternal mortality and improvement in maternal health outcomes?

The present research has triangulated data from various sources and aims to examine the trends in maternal mortality (1997–2017) between EAG and non EAG states in India and explore various household, economic and policy factors that may explain reduction in maternal mortality and improvement in maternal health outcomes. The paper further focusses on evaluating the effect of household wealth status on maternal mortality in India. The results are discussed in the light of various demand side financing initiatives and pro-poor policies designed to reduce maternal mortality and the existing supply side gaps in the healthcare system of India. This paper is timely as India is still a major contributor to the maternal mortality and has initiated a number of demand side financing strategies targeted to poorer sections of the society, aimed at reducing maternal deaths.

## Methods

The primary objective of the study was to estimate the maternal deaths using the different sample surveys conducted in India. The data used in the study were derived from the series of Sample Registration System (SRS) and three round of National Family Health Survey (NFHS) - NFHS-2, 1998–99, NFHS-3, 2005–06 and NFHS-4, 2015–16). NFHS provided micro-level data to understand the determinants of maternal mortality at the individual level whereas SRS was used to analyse trends in maternal mortality, as NFHS was unable to provide nationally representative reliable estimates for the same. The estimates available in SRS for India and major regions of the country based on 3 years’ pooled data have been used to analyse the trend of maternal deaths from 1997 to 2017.

With respect to maternal deaths, household head was enquired about any female (12 years or older) who died during pregnancy, during child birth or within 2 months after the end of pregnancy or childbirth. Using this information available in household file, maternal mortality ratio /100000 live births across the various states of India has been estimated. Household file contains the information of all the household members, including age, sex, marital status, education, collected from the head of the household.

This is calculated for each state using the following formula:

$$ {MMR}_i=\frac{No. of\ maternal\ deaths\ in\ {the\ sample}_i}{Total\  no. of\ live\ births\ in\ {the\ sample}_i}X\ \mathrm{100,000} $$

Here,

*MMR*_*i*_ = Maternal mortality Ratio of state ‘i’

*No. of maternal deaths in the sample*_*i*_ = Number of maternal deaths recorded among the sampled households in state ‘i’

*Total no. of live births in the sample*_*i*_ = Number of total live births recorded among the sampled households in state ‘i’

For the retrospective information regarding maternity history of childbirth and death that took place 5 years prior to the survey, NFHS-4 Kids file was used in order to estimate institutional delivery for states of India. In addition, data on shortfall of health infrastructure and human resources have been taken from Rural Health Statistics, 2005 and 2015 to compare the supply-side infrastructure in India.

Like previous NFHS surveys, NFHS-4 provides information on population, health and nutrition for every State / Union territory in India. All women aged 15–49 years and men aged 15–54 years in the selected sample households were eligible for interviewing. NFHS-4 gathered information from 601,509 households, 699,686 women, and 103,525 men using four different questionnaires [9]. Retrospective information since January 2013 has been collected from head of households regarding the death of any member of the household.

### Analysis

The outcome variable was maternal deaths which has been defined as “1” if a household had any maternal death/deaths and “0” otherwise. Place of residence, education level of household head, region of residence in four categories (EAG, Southern, North East, Rest of India), Wealth Index (WI), households with any insurance coverage, religion, caste, and household size were used as predictors. All the independent variables were categorized to get a sufficient sample of maternal deaths in each category. The details of these variables are available in Supplementary file Table A.1. The analysis for NFHS-4 was restricted to 23,637 households that reported any death in the household since January 2013.

Multivariate analysis was applied to find out the odds of a household to experience a maternal death event. In addition to multivariate analysis, funnel plots were drawn to compare the variation in performance between states on maternal mortality ratio (MMR) per 100,000 live births over time from NHFS-2 (1998–1999) to NFHS- 4 (2015–16). The all India average maternal mortality (indicated by a solid line parallel to the x-axis) was used as a baseline reference. The 95 and 99% confidence bands were constructed and each data point represents the state’s maternal mortality and institutional deliveries. Also, scatter plots were drawn with each data point indicating the state position in relation to maternal mortality and difference poorest and the richest groups simultaneously.

Analysis was carried out using Stata 15.0 software.

## Results

### Trends in maternal mortality and maternal health indicators

**Fig. 1:**
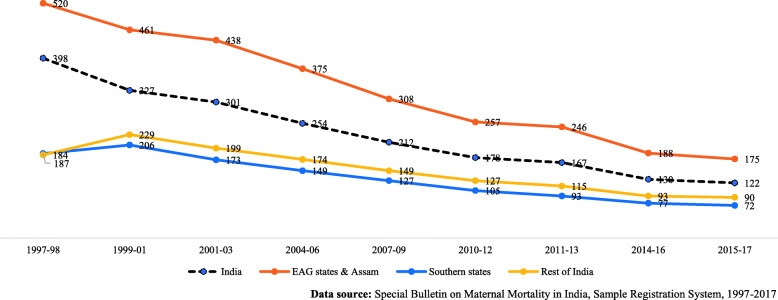
Trend of Maternal Mortality Ratio (Maternal deaths per 100,000 live births) by state groups from 1997 to 2017 in India. Data source: Special Bulletin on Maternal Mortality in India, Sample Registration System, 1997–2017

The historical trend in maternal mortality ratio (MMR) in various groups of states in India from 1997 to 2017 is presented in Fig. 1. MMR has reduced in all regions of the country. In EAG states and Assam, which are considered to be poor performing states in terms of human development and public health, MMR has relatively declined by 66% in the last 20 years. For the better performing states of the Southern region, the relative rate of decline is 62% and for overall India, the relative rate of reduction in MMR in the last two decades is 69%. This improvement is also observed with respect to maternal health indicators.

**Fig. 2:**
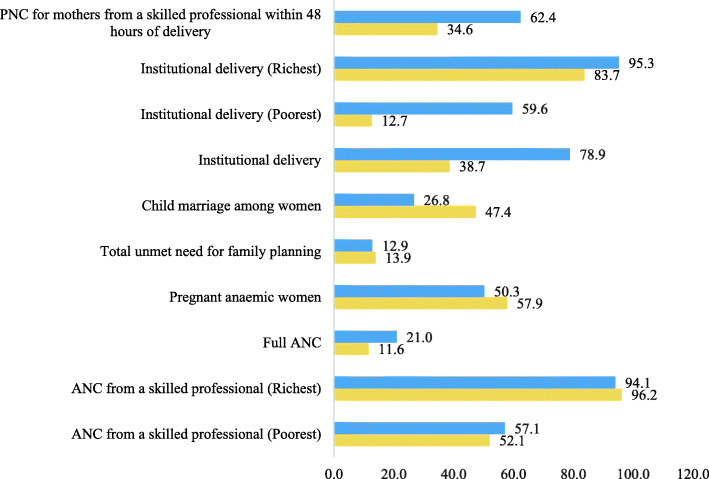
Comparing maternal health care indicators between NFHS 3 (2005–06) to NFHS 4 (2015–16)

Figure 2 provides changes in estimates of various maternal health indicators for women who have at least one live birth in the 5 years preceding the survey periods over the last NFHS decade, 2005–2016. ANC provided by skilled workers for the poorest women has increased by 5%. The gap in ANC services by skilled professional between poorest and richest women has reduced over the study decade. The percentage of women who attained full ANC has increased from 12 to 21%. The percentage of pregnant women with anaemia has reduced by 8% from NFHS 3 to NFHS 4. Total unmet need of family planning has reduced marginally from 14 to 13%. There is a vast decline in marriage of girls below 18 years, from 47% in NFHS 3 to 27% in NFHS 4. The role of institutional delivery in improving maternal health in India is undeniable and widely documented. Institutional deliver has almost doubled from 2005 – 2006 to 2015–16. The rich-poor gap in institutional delivery has halved over the same decade. Post-natal care for mothers within 48 hrs of delivery has almost doubled in the same period.

### Multivariate logistic regression, performance of states, and inequalities

In Table 1 we present the background characteristics of households who experienced maternal death. Table 2 shows the results of multivariate logistic regression on households having any maternal deaths due to various factors in a household. Households having illiterate head of the household had two times higher odds of experiencing maternal deaths in comparison with the households where head of the household was highly educated (*p* < 0.001). The risk of maternal deaths was 78% higher among those households where the head of the household was educated up to primary level in comparison to households where the head was highly educated (*p* < 0.05). The odds of maternal deaths in EAG states was two times in comparison to the Southern states (*p* < 0.05). The poorest households were 80% more likely to experience maternal deaths in comparison to the richest households. However, it is important to note that except for the households belonging to the lowest wealth quintile, the odds ratios were not significant. Further, the households which were not covered under any health insurance scheme were 35% more likely to experience maternal deaths in comparison to those who were covered (*p* < 0.05). It may also be noted that additional multivariate logistic regression (not shown) confirmed that same set of factors namely, illiterate head of households, and households with no health insurance, are two important determinants of adverse maternal health outcomes in both the poor and richer states of India. Religion of the household, and number of members in the household show no significant effect on the maternal deaths in India. The odds of maternal deaths was found to be lower among households belonging to non-SC/ST castes than SC/ST households (*p* < 0.10).

**Table 1:** Background characteristics of those households (HH) which experienced maternal death during 3 years prior to the survey, NFHS-4, 2015–16

Covariates	N	Percentage
**Place of Residence**		
Urban	6095	1.87
Rural	17,542	2.60
**Education level of HH head**		
Higher	2117	1.15
Illiterate	6822	3.34
Primary	4504	2.70
Secondary	10,194	1.87
**Region of residence**		
Southern states	2617	1.13
EAG states	14,011	3.33
North-east	1684	2.42
Rest of India (western+ Northern+west Bengal)	5325	1.59
**Wealth Index**		
Richest	4138	1.26
Poorest	5181	4.10
Poorer	5170	2.30
Middle	4930	2.52
Richer	4218	1.57
**HH any health Insurance coverage**		
Yes	6093	1.49
No	17,544	2.74
**Religion**		
Hindu	18,428	2.37
Non-Hindu	5209	2.45
**Caste**		
SC/ST	7930	3.00
Others	15,707	2.13
**HH Size**		
Less than 5	10,862	2.12
more than or equal to 5	12,775	2.62
Total	23,637	2.38

**Table 2:** Results of multivariate logistic regression of effects of various covariates on maternal deaths, NFHS-4, 2015–16

		95% Confidence interval (CI)	
Covariates	Odds Ratio	Lower Limit	Upper Limit
**Place of Residence**			
Urban	1.00		
Rural	1.04	0.83	1.30
**Education level of HH head**			
Higher	1.00		
Illiterate	2.26***	1.45	3.52
Primary	1.78**	1.13	2.81
Secondary	1.52	0.99	2.34
**Region of residence**			
Southern states	1.00		
EAG states	1.79**	1.25	2.57
North-east	1.50	0.94	2.42
Rest of India (Western+ Northern + West Bengal)	1.05	0.70	1.58
**Wealth Index**			
Richest	1.00		
Poorest	1.80**	1.26	2.59
Poorer	1.37	0.97	1.96
Middle	1.33	0.95	1.89
Richer	1.17	0.83	1.66
**HH any health Insurance coverage**			
Yes	1.00		
No	1.35**	1.10	1.66
**Religion**			
Hindu	1.00		
Non-Hindu	1.19	0.97	1.47
**Caste**			
SC/ST	1.00		
Others	0.84*	0.71	1.01
**HH Size**			
Less than 5	1.00		
More than equal to 5	1.10	0.94	1.30

*p < 0.10; **p < 0.05; ***p < 0.001

**Fig. 3:**
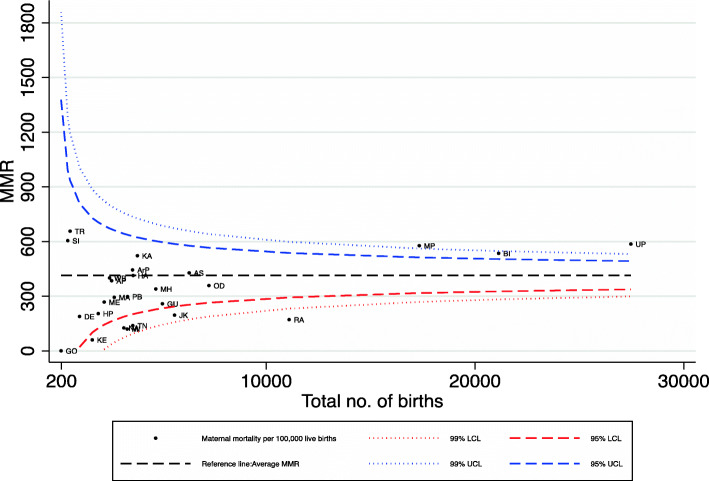
Maternal mortality per 100,000, India, 2015–16(NFHS 4). Note: State codes in Supplementary Table A.2. The following states are merged together for NFHS 4: Andhra Pradesh and Telangana, Madhya Pradesh and Chhattisgarh, Bihar and Jharkhand, Uttar Pradesh and Uttarakhand. UTs are dropped from this figure

In addition to the logistic regression, we analysed the performance of the various states with respect to MMR and the inequalities between the richest and poorest wealth quintiles within the states. Figure 3 provides funnel plots indicating the level of MMR by total births sampled in NFHS 4 (2015–16) and assesses the variation in performance of the various states. This figure helps to identify the states with the lowest and highest MMR, compared with the Indian average (referral line) used as a baseline to compare each state with. The plots closer to the y-axis are states with low numbers of sampled births and those to the right are states with high births. Those states outside the 99% CI or 95% CI can be considered as outliers in terms of their MMR. States which are above the Indian average are the worse-performing states and those below are the better-performing states. It can be observed from Fig. 3 that Madhya Pradesh, Bihar, Uttar Pradesh and Eastern states like Tripura and Assam have performed poorly (State codes in Table A.2). Most of the Southern states are found in the lower side of the funnel plot, indicating low MMR. Analysis over 18 years from NHFS-2 (not shown) reveals that EAG states including Assam have consistently under-performed over the years. It may also be noted that those states which experienced high MMR also had lower institutional deliveries.

**Fig. 4:**
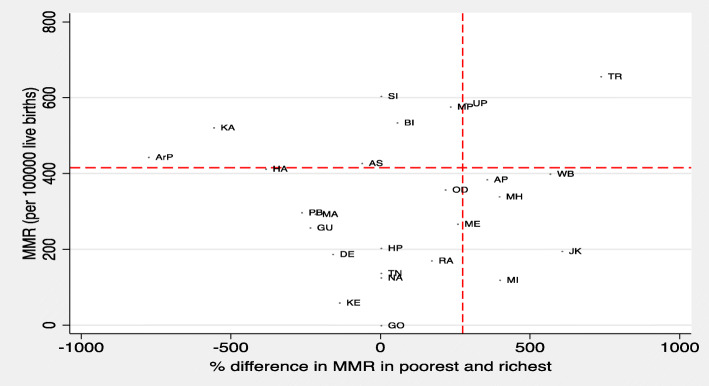
Gap in MMR between poorest and richest households in India, 2015–16. Note: State codes in Supplementary Table A.2, The following states are merged together for NFHS 4: Andhra Pradesh and Telangana, Madhya Pradesh and Chhattisgarh, Bihar and Jharkhand, Uttar Pradesh and Uttarakhand. UTs are not included in this graph for their low sample size

Figure 4 shows the scatterplot of the difference in MMR by top 20% richest and bottom 20% poorest and MMR across various states of India. The states Madhya Pradesh, Tripura and Uttar Pradesh fall in the top right quadrant indicating that these states are poor performers both in terms of having relatively high MMR and also high gap between richest and poorest wealth quintiles. The figure also shows that states like Andhra Pradesh, West Bengal, Maharashtra, Meghalaya, Mizoram, and Jammu & Kashmir with low MMR but high inequality between the poorest and richest quintiles. Odisha and Rajasthan has shown impressive improvement in lowering their MMR and inequality post 2005. States like Goa, Himachal Pradesh, Karnataka, Kerala, Tamil Nadu, and Kerala had consistently performed well and are excellent examples of low MMR with low poorest-richest gap.

### Supply-side constraints: PHC and CHC

For the successful implementation of any maternal health programme, understanding the role of supply-side factors, namely health facilities and human resources is vital. Figure A.1 shows that the gap in shortfall of PHC in rural areas of EAG states and Assam has remained significantly higher than the rest of India. The shortfall has been estimated as the difference between number of PHCs in position in the particular year and the number of PHCs required in the area as per the population norms. The graph implies that in 2018 there was a shortage of 4294 PHCs in EAG states and Assam compared to only 175 in South Indian States. The gap in shortfall of PHCs between the poor performing states (EAG states and Assam) and the better performers (Southern states) has almost doubled from 2004 to 2018.

**Fig. 5:**
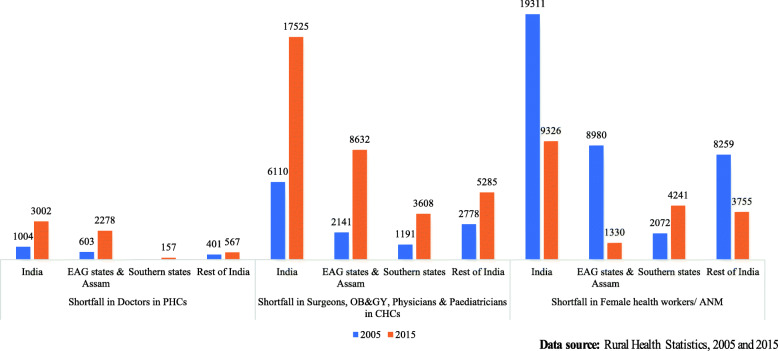
Shortfall of Doctors in PHCs, Specialists in CHCs and female health workers/ ANM in India (in numbers), 2005 and 2015

Finally, Fig. 5 shows shortfall of doctors in PHCs, specialists in CHCs and ANMs from 2005 to 2015. As per the Indian Public Health Standard (IPHS) norms, a minimum of one MBBS doctor should be present in a PHC. Overall, the shortfall in doctors has increased in India. EAG states and Assam contributed majority of the increase in the shortfall of doctors in 2015. As per the revised IPHS norms, the minimum number of specialists required in CHCs is five: General Surgeon, Physician, Obstetrician & Gynaecologist, Paediatrician, and Anaesthetist. The shortfall in specialists in CHCs has tripled from 2005 to 2015. Almost 50% of the aggregate shortfall of specialists in India was found in EAG states and Assam. By IPHS norms, every PHC requires to have one FHW/ANM. The shortfall of FHW/ANM has almost halved in India over the 2005–15 decade. The shortfall has declined in EAG states and Assam too. However, in the Southern states, the shortfall is observed to have doubled over 2005–2015.

## Discussion

India has witnessed a significant decline in its Maternal Mortality Ratio (MMR) in the last 20 years. Approximately 0.14 million women were dying every year on account of complications related to pregnancy and child birth in 1990, which stands reduced by 70% [10]. In addition, India’s share among global maternal deaths has declined significantly from 27.3% in 1990 to 15% [10]. Our findings also confirm that the pace of decline has accelerated since 2000. The relative decline in maternal mortality before 2000 was 32.7% whereas the decline post 2000 till 2015 was 68.7%. Institutional delivery which was just 40% in 2005–6 has almost doubled in a decade to 78.9%.

One of the strengths of this paper is an attempt to triangulate data from various sources in order to understand the trend in maternal mortality with respect to policy initiatives in a chronological order. The present paper shows that the association of household wealth status with maternal mortality is statistically not significant. Our findings suggest that the demand side financing strategies including conditional cash transfers and pro-poor policies designed to address the scourge of maternal mortality in the poorer sections of the society in rural India have an important role to play by reducing the financial barriers in accessing maternal health services. In addition, an important factor in the successful implementation of these cash based incentive schemes is the community-level involvement and the role of community health workers.

A major challenge to policy makers has been to find ways to promote access to maternal services by removing barriers in uptake of these services especially among the poor, and ensure that public subsidies are better targeted to those who need them most [11]. Both these concerns are addressed by demand side financing approaches and therefore, many countries have been experimenting with strategies like conditional cash transfers and use of vouchers to improve access to health services in general and maternal health services in particular [12–20] .

There is evidence to suggest that low access to maternity health services is an important barrier, especially for the poor, in reducing maternal mortality in many low income settings [11, 14, 21]. This holds true even in countries where maternal health care services provided by the public health sector are free at the point of use. Studies have shown that demand side financing like conditional cash transfers and use of vouchers can specifically target and incentivize poor women in order to increase affordability, accessibility, and utilization of maternal health services thereby reducing maternal mortality [14, 20, 22–26] .

The government of India has successfully implemented various conditional cash transfer schemes to remove financial barriers, increase utilization of maternal health services, and promote institutional deliveries [24, 27, 28]. In 1992, the government launched the Child Survival and Safe Motherhood (CSSM), followed by Reproductive and Child Health (RCH) care programmes, I and II, launched in 1997 and 2005 respectively. The Janani Suraksha Yojana (JSY), was launched during the National Rural Health mission, in the year 2005, with the aim to decrease maternal mortality by promoting institutional delivery through conditional cash transfer to women belonging to the poor families. Some of the centrally sponsored schemes are Janani Suraksha Yojana (JSY), Janani Shishu Suraksha Karyakram (JSSK) and Pradhan Mantri Matritva Vandana Yojana (PMMVY), Pradhan Mantri Surakshit Matritva Abhiyan (PMSMA). In addition, as health is a State subject, many States have also implemented financial incentive schemes to improve maternal health outcomes (See Table 3).

**Table 3:** Summary of major Demand side financing schemes for maternal health in India

Scheme name	Launch year	Location	Details	Impact
Dr. Muthulakhsmi Reddy Maternity Benefit scheme	1987	Tamil Nadu	Financial assistance: INR18,000 /− (245 USD) Instalments: 2 Eligibility: minimum 19 years, BPL women in the State Conditionality: restricted for two deliveries and delivered in a government institution	This was one of the pioneering maternity benefit schemes to influence health seeking behaviour away from home care and ensure increased survival of mothers and children [29]. This scheme has been combined with PMMVY in Tamil Nadu.
Sukhibhava	1999	Andhra Pradesh	Integrated with JSY Financial assistance: INR300 (4 USD) for first two deliveries Bank payment: “Sukhibhava” account	This scheme provides evidence that state-level customization of JSY can benefit in addressing wide regional disparities in maternal and child health outcomes.
Janani Suraksha Yojana (JSY)	2005	Central	Eligibility: poor pregnant woman from low performing states (LPS) and high performing states (HPS). All births in LPS and upto 2 live births in HPS. In LPS all pregnant women delivering in Government health centres or accredited private institutions are eligible. In HPS BPL pregnant women, aged 19 years and above are eligible for cash assistance. LPS states: Uttar Pradesh, Uttaranchal, Bihar, Jharkhand, Madhya Pradesh, Chhattisgarh, Assam, Rajasthan, Orissa and Jammu and Kashmir. Rest HPS. Financial assistance: LPS rural areas and urban areas: INR1400 (19.1 USD), INR1000 (13.6 USD) HPS rural areas and urban areas: INR1000 (13.6 USD), INR600 (8.2 USD) Financial incentive to ASHA workers: LPS of INR 600 (8.2 USD) in rural areas and INR 200 (2.7 USD) in urban areas.	Resulted in significant increase in ante-natal care and institutional delivery. Associated with reduction of 3–4 perinatal deaths per 1000 pregnancies and 3 neonatal deaths per 1000 livebirths [27]. Some unintended effects report JSY beneficiaries from LPS are 12% more likely to use contraception, 8% more likely to initiate early breastfeeding and 6% more likely to get their postnatal check-up than mothers from HPS [30].
Janani Evam Bal Suraksha Yojna	2006	Bihar	Integrates cash assistance with institutional care Eligibility: BPL women Financial assistance: INR 1400 (19.1 USD) in rural areas and INR 1000 (13.6 USD) in urban areas paid for birth either in a government or private hospital	ASHA intervention has been effective for the employment of JBSY. It has helped in bridging gaps in the knowledge base of the target communities.
Chiranjeevi Yojana	2006	Gujarat	Eligibility: BPL women Financial assistance: INR 200 (2.7 USD) as financial assistance+ out of pocket transport cost with INR 50 (67 cents) to the attendant. In addition, fixed incentive for private doctors providing gynaecological services. The state pays private doctors a fixed sum of INR6000 (81.8 USD) per 100 births among eligible women living below poverty line (BPL) or belonging to scheduled tribes (ST).	An innovative scheme which increased institutional delivery and provided access to quality maternal care among the poor. Lesser maternal and new-born deaths were observed as a result of this scheme [31].
Sambhav voucher scheme	2007	Uttarakhand/ Uttar Pradesh	Voucher scheme in collaboration with USAID. Institutions are also incentivized so can women avail private facilities. Eligibility for vouchers: Women from BPL family Services include antenatal care, institution birth, post-natal care and family planning services. Accredited private facilities provide services and en-cash the voucher	This is a standalone provider led scheme. Most beneficiaries claimed to have had access to quality health services under this scheme. The effectiveness of the scheme improved due to the involvement of Community Health Volunteers (CHVs). Most urban slum dwellers put their confidence in the scheme mainly due to the CHVs.
Mamata	2011	Odisha	State-sponsored conditional cash transfer maternal benefit scheme to increase utilization of maternal and child healthcare services. Incentive: INR6000 (81.8 USD) over a period of 18 months in instalments. Eligibility: all pregnant and lactating mothers of 19 years and above for the first two births.	Scheme successful in improving antenatal care, exclusive breast feeding, full immunization, etc. A total number of 547,000 mothers were beneficiaries for the financial year 2017–2018 with a budget allocation of INR 3.8 billion (52 million USD). The state is well known for implementing maternal health and nutrition programmes [32].
Janani Shishu Suraksha Karyakram (JSSK)	2011	Central	Eligibility: All pregnant women provided with free delivery including caesarean section in public health facilities. Entitlements: free drugs and consumables; free investigations blood transfusion if needed; free diet; and free return transport. 2014: entitlements extended to all antenatal &post-natal complications of pregnancy; and all sick new-borns and infants (up to 1 year of age).	This scheme promotes free services to pregnant women and sick neonates. In 2019–2020, 9.2 million pregnant women were provided free medicines, 6.2 million free diet, 9.6 million free diagnostics, 3.8 million free transport. Same holds true for sick infants. Significant increase in ante-natal care check-ups and institutional delivery in public facilities due to JSSK [33]; and improved access to level III NICU care among the poor thus reducing preterm mortality rates [34].
Pradhan Mantri Surakshit Matritva Abhiyan (PMSMA)	2016	Central	Comprehensive antenatal services to pregnant women Eligibility: 2nd and 3rd trimester women on the 9th day of every month No. of facilities: 17,217 Private sector involvement: Prime Minister of India appeal to doctors to contribute 12 days in a year.	In the first year of launch, a total of 3,090,270 pregnant women received ANC. More than one crore antenatal check-ups have been conducted till date. While all States/ UTs have made significant efforts to reach out to pregnant women, Rajasthan has largest number of check-ups among the Empowered Action Group States. Includes comprehensive ANC, early identification and follow-up of high risk pregnancies. Identifying high risk pregnancies is necessary step in reducing avoidable maternal and infant deaths [35]. To detect high risk pregnancies, 84 lakh haemoglobin tests, 55 lakh HIV tests, 41 lakh tests for gestational diabetes, 33 lakh tests for syphilis and more than 15 lakh ultrasounds have been performed. Over 5.50 lakh pregnant women were identified as high risk pregnancies and referred to a specialist or a higher health facility for appropriate care.
Pradhan Mantri Matrutva Vandan Yojana (PMMVY)	2017	Central	Implementation: Integrated Child Development Services. Financial assistance: INR 5000 (68.2 USD) in Three Instalments for the First Live Child.	From 2017 to 2020, 1,36,80,531 beneficiaries [36]. However, this scheme is criticized for excluding women with more than 1 children. A survey showed that only 22% pregnant and lactating women were covered under this scheme [37].

1 USD = 73.33 INR as on 8th January, 2021 obtainable from https://www.imf.org/external/np/fin/data/rms_mth.aspx?SelectDate=2020-11-30&reportType=REP

JSY was launched in 2005 by Ministry of Health and Family Welfare under the umbrella of National Rural Health Mission aimed to decrease maternal mortality by promoting institutional delivery. JSY provides incentives in the form of conditional cash transfer where money aids in changing the conduct of women who avail themselves of three antenatal check-ups and opt for institutional delivery [38]. However, eligibility criteria and monitory incentive amount varies as per the performance of the state. Accredited Social Health Activists (ASHAs) and Auxiliary Nurse Midwives (ANMs) play an important role in the successful implementation of the JSY [38]. There is a direct relation between incentivizing (like JSY) and increased use of health facilities for maternal and child health [38, 39]. JSY has expanded over the years, both in terms of coverage and budget, and has contributed significantly to the improvements in maternal health outcomes in India. This scheme covered fewer than 1 million mothers in 2005 and now covers over 10 million mothers. Similarly, its expenditure has increased from less than 0.4 billion INR (5.5 million USD) to about 18 billion INR (245 million USD) currently [10].

Subsequently, building on JSY, another centrally implemented scheme JSSK was launched in 2011, which mainly focused on reducing out of pocket expenditure among pregnant women [40]. As a result of JSY & JSSK, utilization of public health services has increased significantly by pregnant women over the years. More recently, Pradhan Mantri Surakshit Matritva Abhiyan (PMSMA) was launched in 2016 which entitles all pregnant women to the comprehensive package including quality antenatal care, free of cost in the public health facility. In just 13 months, PMSMA successfully achieved 13 million antenatal check-ups and resulted in diagnosis of 0.65 million high-risk pregnancies. In addition to the centrally sponsored schemes, various states have also implemented their own financial incentive schemes (See Table 3). Various studies too support our conclusion that conditional cash transfers removed financial barriers to access, improved utilisation of maternal health services, and promoted institutional deliveries in India [25, 27, 29, 38–40].

Our results also indicate the importance of ancillary factors and broader social determinants of maternal health in contributing to the reduction in maternal mortality and morbidity. For example, India has witnessed increase in contraceptive use resulting in decrease in unmet need. Increasing contraceptive use contributes to a decline in high parity births that elevate the risk of maternal mortality [41]. Similarly, poor nutritional status and Vitamin A deficiency among pregnant women can lead to chronic conditions like eclampsia, preeclampsia, ante-partum and post-partum haemorrhage, thus being a contributory factor for maternal mortality [42]. Our findings suggest significant reduction in nutritional deficiency anaemia among women over the years. India has also witnessed reduction in child marriages among women. Early marriages have a higher risk of intimate partner violence, HIV/STIs, depression, and contribute to maternal morbidity and mortality [43]. Also, female literacy has dramatically increased from 32.3 in 1991 to 65.8 in 2018 [44]. Finally, households below poverty line have halved from 45.3 in 1993 to 21.9 in 2012 [45]. In terms of HDI, India’s value increased from 0.43 to 0.65 from 1990 to 2018 and India now ranks at 129 out of 189 countries in 2018 [46]. Thus, as a result of these conditional cash transfer schemes, there has been improved utilization of maternal health services, including births in health facility, which have contributed significantly to the reduction in the share of MMR. In addition, targeting ancillary and broader social determinants of maternal health along with various social marketing strategies has further accelerated the trend of declining maternal mortality in India.

Although India is moving in the right direction, and has achieved considerable decline in maternal mortality, it needs further intense efforts. Compared to other countries in the region, India is only better than Nepal, Bhutan and Afghanistan in terms of maternal mortality and at par with Bangladesh and Pakistan. It still lags behind other regional countries like Indonesia, Thailand, Sri Lanka and China. In addition, as our analysis reveals, the gains in terms of maternal mortality have been unevenly distributed in India. There exits huge inequalities in maternal mortality and other maternal health outcomes between EAG states and socio-economic groups within the states. For example, maternal mortality varies from less than 70/100000 live births in states like Kerala, Maharashtra, Tamil Nadu to over 200 in EAG states like UP and Assam. In addition, our multivariate logistic analysis reveals that women belonging to poorest households, illiterate or educated below secondary schooling, without any health insurance coverage and residing in EAG states have a significant higher maternal mortality. Although the female literacy has increased for India as a whole, the gap between the EAG and non-EAG states is over 13%.

Policy makers need to prioritise underperforming states and socio-economic groups within the states where intense efforts need to be expanded. In addition, it is necessary to address supply-side factors hand in hand with demand-side measures. Primary Health Centres (PHCs) which are a backbone for providing basic health services including maternal health services to the rural population, have been neglected historically among the EAG states and Assam. The gap in terms of PHC shortfall between these states and Southern states has been observed since 2004, and this gap has dramatically increased over the years. The same holds true with respect to shortfall in terms of human resource availability i.e. shortfall of PHC doctors; Obstetricians, Physicians and Paediatricians have drastically increased in EAG states from 2005 to 2015.

A major concern still remains in terms of inadequate health care financing [47] as India’s government health expenditure as a percentage of GDP is a meagre 1.28%. This is one of the lowest government health expenditures among countries in the region including Nepal, Sri Lanka, Thailand, and Indonesia. Such low public health expenditures coupled with high out of pocket payments (67.8% of total health expenditure) need to be addressed before India makes further progress. Together, demand-side and supply-side measures mediated by contextual factors are likely to improve maternal health outcomes in underperforming states of India.

## Conclusions

India has been able to drastically reduce its maternal mortality and improve maternal health outcomes in recent years. This impressive performance can be attributed to the various policies and cash incentive schemes launched during the launch of National Rural Heath Mission in 2005. Our findings suggest that Wealth Index was not found to be statistically significant in either group of states, which supports our conclusion that any effective pro-poor policy aims to minimise the impact of household’s wealth status on adverse maternal health outcomes by reducing the financial barrier to access maternal health services. In addition to the more targeted approach, India has also attempted to address broader social determinants of maternal health which have contributed to improving maternal outcomes. However, the distribution of these gains is uneven across states and socio-economic groups. Along with demand side financing schemes, supply side measures are necessary to accelerate the gains in maternal outcomes in the underperforming states.

## Supplementary Information

**Additional file 1:** **Table A.1.**. Variables used for analysis. This Table provides the details of the variables used for the analysis in the manuscript. **Figure A.1.** Trend in shortfall in primary health care facilities in rural India, 2004–2018. This figure provides the shortfall in PHC facilities among EAG States including Assam, Southern States and rest of India. **Table A.2.** State codes. The codes for states of India that have been used in the analysis are provided in this table.

## Data Availability

The datasets analysed during the current study are from National Family Health Survey (NFHS) for India. The data is freely available from the DHS website The DHS Program - India: Standard DHS, 2015–16 Dataset.

## Abbreviations

ANC: Ante-natal care
ANM: Auxiliary Nurse midwives
ASHA: Accredited Social Health Activist
CHC: Community Health Centre
CSSM: Child Survival and Safe Motherhood
FHW: Female Health worker
HDI: Human Development Index
IPHS: Indian Public Health Standard
JSY: Janani Suraksha Yojana
JSSK: Janani Shishu Suraksha Karyakram
MDGs: Millennium Development Goals
MMR: Maternal Mortality Ratio
NFHS: National Family Health Survey
PHC: Primary Health Care Centre
PMMVY: Pradhan Mantri Matritva Vandana Yojana
PMSMA: Pradhan Mantri Surakshit Matritva Abhiyan
RCH: Reproductive and Child Health
SDGs: Sustainable Development Goals
SRS: Sample Registration System
UNICEF: United Nations Children’s Fund,
WHO: World Health Organization
