# Supplementary material for: Pro-poor policies and improvements in maternal health outcomes in India
Source: BMC Pregnancy Childbirth. 2021 May 19;21:389. doi: 10.1186/s12884-021-03839-w (PMC8135986; doi:10.1186/s12884-021-03839-w)
Supplement: Supplementary file 1 — Additional file 1: Table A.1.. Variables used for analysis. This Table provides the details of the variables used for the analysis in the manuscript. Figure A.1. Trend in shortfall in primary health care facilities in rural India, 2004–2018. This figure provides the shortfall in PHC facilities among EAG States including Assam, Southern States and rest of India. Table A.2. State codes. The codes for states of India that have been used in the analysis are provided in this table. [file 12884_2021_3839_MOESM1_ESM.docx]

**Supplementary Figures and Tables**

**Table A.1. Details of the variables used for analysis**

| **Variable** | **Categories** |
| --- | --- |
| **Place of residence** | Urban |
|  | Rural |
| **Education level of HH head** | No education |
|  | Primary |
|  | Secondary |
|  | Higher |
| **Region of residence*** | EAG** |
|  | Southern |
|  | North East |
|  | Rest of India ( Northern, Western and West Bengal) |
| **Wealth Index** | Poorest |
|  | Poorer |
|  | Middle |
|  | Richer |
|  | Richest |
| **Caste of Household** | Scheduled Caste (SC) / Scheduled Tribe (ST) |
|  | Others (do not belong to Scheduled caste/ Scheduled Tribe) |
| **Religion** | Hindu |
|  | Non- Hindu ( Muslims, Christians, Jains, Sikhs, Buddhist/neo-Buddhist, Jewish, Parsis, no religion, others) |
| **Households with any insurance coverage** | No (No Health Insurance cover) |
|  | Yes (Has Health Insurance cover) |
| **Household Size** | Less than five |
|  | More than equal to five |

*In order to get sufficient sample of maternal deaths in each category, states and UTs of India have been divided into four broad subgroups namely EAG (empowered action group) states comprising of “Bihar, Chhattisgarh, Jharkhand, Madhya Pradesh, Odisha, Rajasthan and Uttarakhand”; Southern states comprising of “Andaman & Nicobar Island, Andhra Pradesh, Karnataka, Kerala, Lakshadweep, Pondicherry, Tamil Nadu and Telangana”; North East was made from “Arunachal Pradesh, Assam, Manipur, Mizoram, Meghalaya, Nagaland, Sikkim and Tripura” and Rest of India (ROI) region was made from remaining states.

** The states are called EAG as they lag behind in the demographic transition and have the highest infant, under 5, and maternal mortality rates in the country.

**Figure A.1. Trend in shortfall in primary health care facilities in rural India, 2004-2018**

**Note:** The requirement is calculated using the prescribed norms on the basis of total and tribal population in rural India from Census, 2001 (for 2004, 2006) and 2011 (for 2016 and 2018). All India shortfall estimates is calculated by ignoring existing surplus in some states.

Data source: Rural Health Statistics, Health and Family Welfare statistics

**Table A.2. State codes**

| Andaman and Nicobar Islands | AN |
| --- | --- |
| Andhra Pradesh | AP |
| Arunachal Pradesh | ArP |
| Assam | AS |
| Bihar | BI |
| Chandigarh | CG |
| Chhattisgarh | CH |
| Dadra and Nagar Haveli | DN |
| Daman and Diu | DD |
| Delhi | DE |
| Goa | GO |
| Gujarat | GU |
| Haryana | HA |
| Himachal Pradesh | HP |
| Jammu and Kashmir | JK |
| Jharkhand | JH |
| Karnataka | KA |
| Kerala | KE |
| Lakshadweep | LD |
| Madhya Pradesh | MP |
| Maharashtra | MH |
| Manipur | MA |
| Meghalaya | ME |
| Mizoram | MI |
| Nagaland | NA |
| Odisha | OD |
| Puducherry | PU |
| Punjab | PB |
| Rajasthan | RA |
| Sikkim | SI |
| Tamil Nadu | TN |
| Telangana | TE |
| Tripura | TR |
| Uttar Pradesh | UP |
| Uttarakhand | UT |
| West Bengal | WB |
